# An Ilomastat-CD Eye Drop Formulation to Treat Ocular Scarring

**DOI:** 10.1167/iovs.16-21377

**Published:** 2017-12

**Authors:** Abeer H. A. Mohamed-Ahmed, Alastair Lockwood, He Li, Maryse Bailly, Peng T. Khaw, Steve Brocchini

**Affiliations:** 1 UCL School of Pharmacy, London, United Kingdom; 2 UCL Institute of Ophthalmology, London, United Kingdom; 3 The National Institute for Health Research (NIHR) Biomedical Research Centre at Moorfields Eye Hospital NHS Foundation Trust, London, United Kingdom

**Keywords:** ilomastat, antiscarring, ocular drug delivery, cyclodextrin, solubilization

## Abstract

**Purpose:**

The purpose of this study was to develop a topical matrix metalloproteinase inhibitor preparation for antiscarring therapy.

**Methods:**

The broad spectrum matrix metalloproteinase inhibitor ilomastat was formulated using 2-hydroxypropyl-β-cyclodextrin in aqueous solution. In vitro activity of ilomastat-cyclodextrin (ilomastat-CD) was examined using fibroblasts seeded in collagen. Permeation of ilomastat-CD eye drop through pig eye conjunctiva was confirmed using Franz diffusion cells. Ilomastat-CD eye drop was applied to rabbit eyes in vivo, and the distribution of ilomastat in ocular tissues and fluids was determined by liquid chromatography-mass spectroscopy.

**Results:**

The aqueous solubility of ilomastat-CD was ∼1000 μg/mL in water and 1400 μg/mL in PBS (pH 7.4), which is greater than ilomastat alone (140 and 160 μg/mL in water and PBS, respectively). The in vitro activity of ilomastat-CD to inhibit collagen contraction in the presence of human Tenon fibroblast cells was unchanged compared to uncomplexed ilomastat. Topically administered ilomastat-CD in vivo to rabbit eyes resulted in a therapeutic concentration of ilomastat being present in the sclera and conjunctiva and within the aqueous humor.

**Conclusions:**

Ilomastat-CD has the potential to be formulated as an eye drop for use as an antifibrotic, which may have implications for the prevention of scarring in many settings, for example glaucoma filtration surgery.

Matrix metalloproteinases (MMPs) are proteolytic enzymes that are essential in wound healing, with roles in inflammation and extracellular matrix remodeling.^1^ Diseases related to uncontrolled extracellular matrix destruction with upregulated MMP expression include inflammatory arthritis and atherosclerosis.^2,3^ Broad spectrum inhibitors of MMP (MMPi) have shown promise as modulators of healing with in vitro studies showing the inhibition of fibroblast functions such as migration, collagen production, and collagen contraction.^4,5^ Furthermore, these are nontoxic to ocular cells in vivo^6,7^ and in vitro.^4,5^ Preclinical studies have shown that the synthetic broad spectrum MMPi ilomastat can inhibit conjunctival scarring after glaucoma filtration surgery in rabbits,^6,7^ in lens capsules following simulated cataract surgery,^8^ and in models of vitreoretinal contraction.^9^

The topical administration of ilomastat following glaucoma surgery would potentially avoid the need for subconjunctival injections. Ilomastat is poorly soluble in water (∼140 μg/mL at 25°C)^10^ and 161 μg/mL in balanced salt solution at 35.5°C.^11^ Attempts to prepare sodium salts of ilomastat failed because ilomastat is a weak hydroxamic acid with a relatively high pKa = 8.9^12^ that precluded salt formation at physiological pH (unpublished data). Solubilization of ilomastat upon addition of sodium hydroxide (ilomastat/NaOH, 1:2 molar ratio) was observed, but the lyophilized powder of this solution was not soluble in water (formation of suspension) (unpublished data).

Ilomastat has been reported to reduce corneal damage caused by alkali burn in rabbits.^13^ Ilomastat was dissolved in HEPES buffer containing 0.1% dimethylsulfoxide (DMSO) and then administered topically to treat corneal damage in the rabbits.^13^ The same vehicle was used to solubilize ilomastat in a phase 1 clinical trial in healthy volunteers.^14^ Both safety and efficacy of ilomastat were shown in phase I/II trials in patients with bacterial keratitis induced corneal damage using topical ilomastat, but development was discontinued because the company (GlycoMed) merged with another company with different interests.^14,15^ Increasing the amount of ilomastat within an eye drop formulation would potentially result in an adjunct therapy to treat fibrosis on the ocular surface and within the subconjunctival space after glaucoma surgery.

Cyclodextrins (CDs) are hydrophilic cyclic oligosaccharides that are able to form water soluble complexes with some lipophilic drugs.^16^ CDs have been used preclinically to formulate eye drops with drugs such as dorzolamide,^17^ cilostazol,^18^ natamycin,^19^ hydrocortisone,^20^ cyclosporine A,^21^ antihypertensive drugs,^22^ ciprofloxacin,^22^ tropicamide,^23^ prostaglandins,^24^ pilocarpine hydrochloride,^25^ and thalidomide.^26^ Several CD-based eye drops have been evaluated clinically, for example, latanoprost and dexamethasone,^27–30^ and several CD eye drop formulations have been registered for clinical use, for example, chromaphenicol (Clorocil, registered in Europe), diclofenac (Voltaren Ophthalmic, registered in Europe), and indomethacin (Indocid, registered in Europe).^31^ In most of these formulations, the percentage of CD used for solubilization of the drugs was in the range of 10% to 30% wt/vol.^17,18,27,32^ The complex forming characteristics of ilomastat with CD are not known, so we decided to investigate whether a soluble ilomastat-CD complex could be prepared and then used as an eye drop to treat the conjunctiva. It was hoped that complexation of ilomastat with CD, to give ilomastat-CD, would improve the ocular bioavailability of ilomastat via a soluble CD complex so that ilomastat could permeate through the conjunctiva.

## Materials and Methods

### Materials

Ilomastat (388.6 Da) was purchased from Ryss (Union City, CA, USA). (2-Hydroxypropyl)-β-CD (molecular weight 1380 Da) was purchased from Sigma Aldrich Corp. (332593-5G; Irvine, Scotland, UK). Healon GV (1.4% hyaluronic acid [HA] sodium salt in physiological buffer) was purchased from Moorfields Pharmaceuticals (London, UK). Acetonitrile (HPLC grade) and formic acid were purchased from Fisher Scientific UK Ltd. (Loughborough, UK).

### Solubility Studies

Phase solubility studies were conducted to determine the amount of CD required to solubilize ilomastat. An excess amount of ilomastat (5 mg) was suspended in water (1 mL) and stirred for 30 minutes. CD was added slowly to ilomastat suspension over 10 minutes at concentrations of 0.5%, 1%, 5%, 10%, 15%, and 20% wt/vol. The mixture was left to stir overnight. The uncomplexed ilomastat was removed by filtration (0.22 μm polyethersulfone [PES]). The concentration of ilomastat in the filtrate was determined by HPLC.

### Chromatographic Determination

Ilomastat was quantified using HLPC (Agilent 1200 series HPLC system, Agilent Technologies, London, UK) fitted with a Synergi RP Phenomenex 4-μm, 15-cm C18 column and equipped with an autosampler, a degasser, and two SL bin-pumps. A flow rate of 1 mL/min was used with 0.1% trifluoroacetic acid in water and acetonitrile as eluents A and B, respectively, with a linear gradient from 80% A to 70% B in 17 minutes. The detection wavelength was 280 nm.

### Preparation of Ilomastat-CD Solution

The percentage of CD that gave the highest ilomastat solubility was selected from the phase solubility studies. Ilomastat (4 mg, 0.00001 mol) was suspended in PBS (2 mL) followed by the slow addition of CD powder (400 mg, 0.00029 mol) (molar ratio of ilomastat/CD is 1:29). The mixture was left to stir overnight. Benzalkonium chloride (BAC) (0.8 mg) was then added to the mixture, and the mixture was filtered (0.22 μm PES). Ilomastat concentration in the filtrate was determined by HPLC (equilibrium concentration was 1.43 ± 0.01 mg/mL [*n* = 4]) and adjusted to 1 mg/mL in PBS [pH 7.4]).

The sodium salt of HA (Healon GV) was used to increase ilomastat residence time. Healon GV (1.4% wt/vol HA, 0.55 mL) was dissolved in PBS (1.375 mL) to prepare HA 0.4% wt/vol. HA solution (0.4% wt/vol, 1 mL) was added to 1 mL ilomastat-CD solution (1 mg/mL) to give clear solution. The final concentration of ilomastat was 0.5 mg/mL (0.05% wt/vol) in a clear solution containing CD (10% wt/vol), HA (0.2% wt/vol), and BAC (0.02% wt/vol) in PBS (pH 7.4).

### Permeation Study

The conjunctival permeation studies were conducted using Franz diffusion cells at 35°C. The conjunctivas harvested from porcine eyes were placed on filter paper and then placed between donor (volume = 1 mL) and receptor (volume = 2 mL) chambers within 20 minutes after excision, with an available area for diffusion of 1 cm^2^. The receptor chamber was filled with PBS buffer (pH 7.4, 2 mL). Ilomastat was applied onto the conjunctiva at a dose of 50 μg (100 μL) as either of ilomastat-CD HA or ilomastat-CD solution (0.5 mg/mL ilomastat) or 500 μL ilomastat-PBS (100 μg/mL). At time intervals of 10, 30, 60, 90, 120, 180, and 240 minutes, 1 mL of sample was withdrawn from the receptor chamber and an equal amount of PBS buffer was added to maintain the original volume. Each experiment was run in triplicate, and drug concentrations were determined by HPLC.

### Cell Culture

Human Tenon fibroblast cells (HTFs) were maintained in fibroblast culture medium composed of Dulbecco's modified Eagle's medium (DMEM) supplemented with 10% (vol/vol) fetal bovine serum (FBS), 2 mM l-glutamine, 100 IU/mL penicillin, and 100 g/mL streptomycin (all from Gibco Life Technologies, Paisley, Scotland, UK) at 37°C with 5% (vol/vol) CO_2_ in air. The medium was changed every 3 to 4 days until cells reached confluence. The cells were passaged at a ratio of 1:4. Cultures were used between passages 3 and 10 for the gel contraction experiments.^33^

### Gel Contraction Assay

The free-floating collagen lattice model was used to assess inhibition of gel contraction by ilomastat.^33,34^ HTF cells (7 × 10^4^ cells/mL) were seeded in collagen type-1 lattice (150 μL) in a Matteck dish. The stock solution of ilomastat was prepared in DMSO. Ilomastat-CD solution (20% CD) in PBS was filtered and sterilized (0.22 μm). Stock solutions of ilomastat (DMSO) and ilomastat-CD (PBS) were diluted in DMEM (FBS, 10% vol/vol) to 0.01, 1, 10, and 100 μM ilomastat. The drug solutions were added to the lattice (2 mL) at each individual concentration and incubated at 37°C. The gels were imaged daily from day 0 to day 7, and the percent contraction was calculated based on the decrease in gel area using ImageJ software (http://imagej.nih.gov/ij/; provided in the public domain by the National Institutes of Health, Bethesda, MD, USA). The shrinkage of the gel was considered as an indicator of fibrosis activity of the cells.^4,33,34^ Each formulation was tested in triplicate.

### In Vivo Distribution of Ilomastat

Six female New Zealand White rabbits (2–2.4 kg, 12–14 weeks old; Harlan Laboratories Ltd., Shardlow, UK) were studied for ocular drug distribution post eye drop application. These experiments were performed in accordance with the ARVO Statement for the Use of Animals in Ophthalmic and Vision Research. Ilomastat-CD solution (20% CD without HA) (100 μL) was topically applied to right rabbit eye using a 1-mL insulin syringe. After 4 hours, the rabbit eyes were rinsed with water and removed. Ocular tissues and fluids were isolated immediately after removal of rabbit eyes and frozen quickly before being stored at −80°C until analyzed.

#### Extraction of Ilomastat From Ocular Fluids

Ocular fluid (aqueous humor or vitreous) (200 μL) was spiked with internal standard marimastat (10 μL, 1 μg/mL). Proteins were precipitated by adding twice the volume of methanol (400 μL), vortexed for 5 minutes, then centrifuged at 3396*g* for 20 minutes. Supernatant was removed and dried by evaporation at 40°C under nitrogen. The dried material was then reconstituted in 100 μL acetonitrile (50% in water) containing 0.1% formic acid and then analyzed by LC-MS.

#### Extraction of Ilomastat From Ocular Tissues

Ocular tissues (sclera, conjunctiva, and cornea) were dissected, then frozen quickly in dry ice and stored at −80°C. Tissues were lyophilized, and the weight of the dry tissue was recorded. PBS (pH 7.4) was added to ocular tissues (800 μL) and incubated for 1 hour at 55°C. Proteinase K (1 mg/mL in PBS) equal volume was added to tissues and then incubated at 55°C for 4 hours with shaking (1.77*g*). Tissue homogenates were vortexed for 5 minutes. Proteins were precipitated by adding double the volume of methanol followed by vortexing for 5 minutes. Supernatant was then removed. An equal volume of diethyl ether was added to the supernatant to precipitate fatty acids. Solutions were mixed and centrifuged at 3396*g* for 20 minutes. Supernatants were removed and concentrated under vacuum to 1 mL. Distilled water (2 mL) was then added to the concentrated solution. Liquid-liquid extraction was then conducted by adding 9 mL ethyl acetate to the aqueous solution followed by vigorous mixing. The extraction solutions were left to mix for 48 hours. Then the ethyl acetate phase was carefully collected using a glass pipette and dried by evaporation at 40°C under nitrogen. The dried material was then reconstituted in 100 μL acetonitrile (50% in water) containing 0.1% formic acid, then analyzed by liquid chromatography-mass spectroscopy (LC-MS).

### LC-MS Analysis

An Agilent Zorbax Eclipse Plus C18 (3.5 μm, 50 × 2.1 mm; Agilent Technologies, London, UK) column was used with a mobile phase consisting of solvent A (0.1% formic acid in water) and solvent B (0.1% formic acid in acetonitrile), and gradient elution was used (Supplementary Table S1). Flow rate was 200 μL/min, and run time was 7 minutes. Ilomastat and marimastat eluted at 3.10 and 2.96 minutes, respectively. Triple quadrupole LC-MS (TSQ Quantum Access; Thermo Scientific, London, UK) was used. SRM scan for both parent compound and ion products mass (mass-to-charge ratio, m/z) was conducted and recorded over masses of 50 to 450. In the first scan, the parent ilomastat and marimastat masses [M + H] were 389.36 and 332.31, respectively. In the second scan, ionization of the parent compounds was conducted. The most abundant fragments were selected for ilomastat m/z of 356.06 and 227.02 and for marimastat m/z of 301.03 and 273.03. Collision energy used was 12 and 10 for ilomastat and marimastat, respectively. Quantification of ilomastat in ocular tissues and fluids was accomplished by constructing a calibration curve of area under the curve ratio (drug/internal standard) versus concentration. The area under the curve was calculated using ilomastat m/z 356 and marimastat m/z 301 for ocular fluids. For ocular tissues the peak used to quantify ilomastat was m/z 227. The data were expressed in average nanomolars ± standard error of the mean for ocular fluids and average picogram per milligram of tissue ± standard error of the mean.

## Results

### Formation and Solubility of Ilomastat-CD

**Figure 1:**
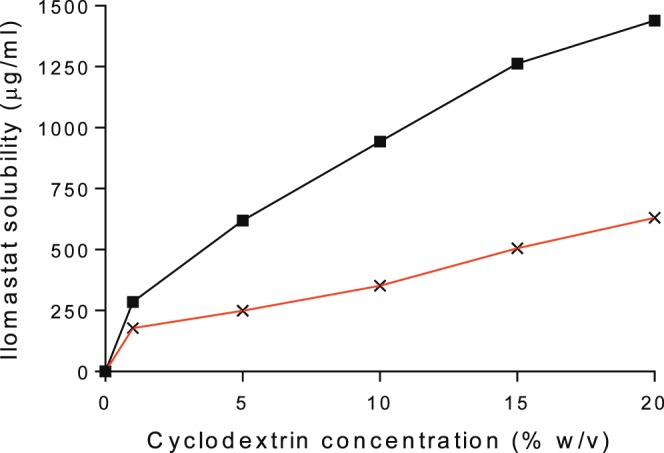
Solubility of ilomastat-CD (CD is 1%–20% wt/vol) in PBS (pH 7.4) (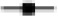) and water at room temperature (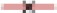) after stirring for 1 day (n = 3).

The solubility of ilomastat in water and PBS was proportional to the amount of CD that was added (Fig. 1). The highest solubility of ilomastat in water was achieved by using CD 20% wt/vol. The solubility after gentle stirring of ilomastat/CD mixture was 630.1 μg/mL after 1 day and increased to 1029.1 μg/mL after 2 days. There was no change in the ilomastat HPLC profile. Poly(vinylpyrrolidone) (PVP; 0.25%–1.0% wt/vol), known to improve the solubility of some CD complexes through the formation of a ternary complex with drug-CD complex,^35,36^ was examined in an effort to further improve the solubility of the ilomastat-CD complex. However, the addition of PVP to ilomastat-CD (20% wt/vol) only slightly increased the solubility of ilomastat in water (1137.6 μg/mL) after being stirred for 2 days (Supplementary Fig. S1). Alexanian et al.^36^ reported comparable findings where PVP did not enhance the solubility of nimesulide-CD complex. PVP solubility enhancement appears to depend considerably on the drug.^36,37^

Our aim was to improve the solubility of ilomastat at pH 7.4, which is the optimum physiological pH for topical ophthalmic administration. A mild and relevant solution such as PBS at pH 7.4 can be used to administer ilomastat to treat irritated, injured eyes to aid the healing process. Our strategy is supported by other studies that describe the use of CD to improve drug solubility at a fixed pH that would be used in the final formulation.^18,38,39^ Better ilomastat solubility was achieved as the CD complex in PBS (1439.93 ± 5.96 μg/mL) using CD at a concentration of 20% wt/vol after 1 day of gentle stirring of the ilomastat/CD mixture (Fig. 1). From these solubility studies, PBS (pH 7.4) was found to be the best solvent for preparation of topical ilomastat-CD complex solution using 20% wt/vol CD.

### Permeation Studies

**Figure 2:**
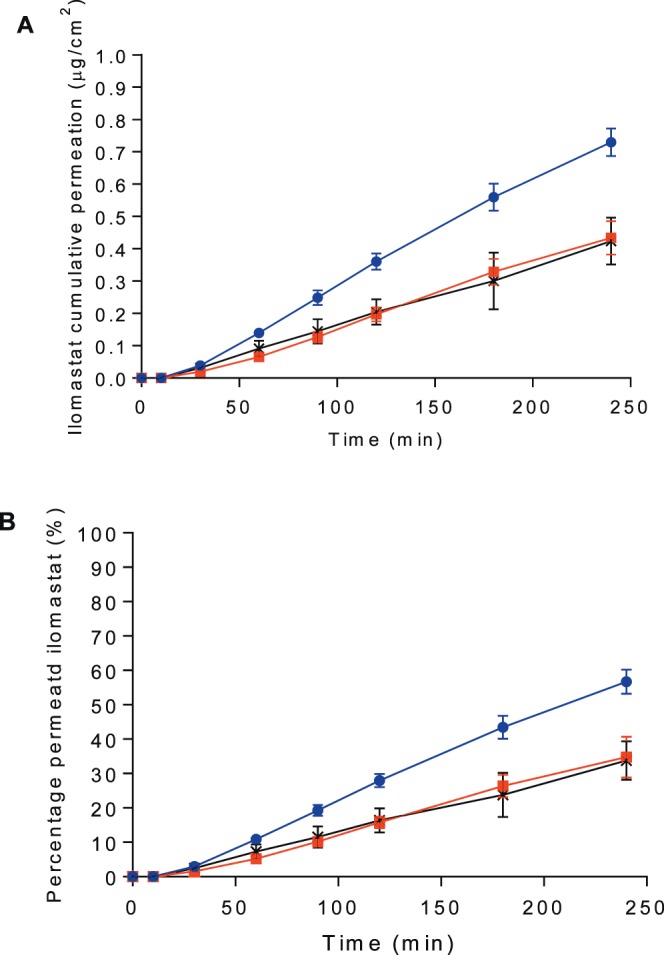
Permeation profile of ilomastat-CD (50 μg ilomastat) in pig's conjunctiva in vitro at 35°C. (A) Ilomastat cumulative permeation (μg) per surface area of pig's conjunctiva (cm^2^). (B) Percentage of ilomastat initial dose that permeated through pig's conjunctiva. Ilomastat-CD containing 0.2% HA wt/vol (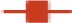), ilomastat-CD (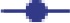) and pure ilomastat in PBS solutions (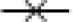). Data are average ± standard deviation (n = 3).

The permeation of ilomastat through pig conjunctiva ex vivo was examined at an ilomastat dose of 50 μg (100 μL ilomastat-CD, 500 μg/mL). Ilomastat in PBS was included as control at a concentration of 100 μg/mL (500 μL). The permeation of ilomastat through pig conjunctiva increased with incubation time (Fig. 2). HA was also evaluated as an excipient at a concentration similar to that used in an existing commercial artificial tear product^40^ in an effort to develop ilomastat-CD formulation with more optimal properties on the ocular surface. Several studies report that HA can be used to increase the residence time of a drug on the surface of the eye,^41,42^ which could be beneficial to increase the concentration of ilomastat in the subconjunctival space. Although the permeation profiles are similar, the ilomastat-CD complex without HA displayed higher permeation than ilomastat-CD containing 0.2% HA and pure ilomastat (Fig. 2). Complexation of ilomastat with CD helped to increase the permeation of ilomastat through the conjunctiva. In contrast, the presence of HA in the ilomastat-CD solution eliminated the enhanced penetration effect of CD. The reduced permeation of ilomastat-CD containing 0.2% HA may be due to the need for ilomastat to diffuse through the viscous HA solution, so we decided to use ilomastat-CD solutions only as eye drop without addition of HA.

### In Vitro Efficacy of Ilomastat-CD

**Figure 3:**
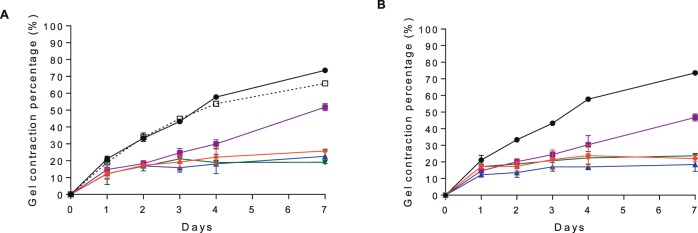
Effect of ilomastat on ocular fibroblast-mediated collagen contraction at different concentrations (0.01 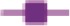, 1 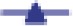, 10 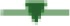, and 100 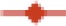 μM). (A) Pure ilomastat solubilized in DMSO and (B) ilomastat-CD complex. DMSO (% vol/vol in DMEM) (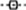) and DMEM blank (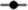) were included as control. Data are average ± standard deviation (n = 3).

An in vitro assay utilizing HTFs seeded on collagen and then measuring collagen contraction is often used to evaluate the potential antifibrotic activity of candidate drugs in vitro.^4^ Exposure of both ilomastat and ilomastat-CD significantly inhibited collagen gel contraction over a 7-day incubation period (Fig. 3). Ilomastat-CD displayed inhibition of gel contraction similar to ilomastat at all concentrations that were examined. At day 7, at an ilomastat concentration range of 1 to 100 μM, ilomastat-CD displayed 18.42% ± 4.09% to 23.72% ± 1.22% gel contraction, which is similar to pure ilomastat (19.09% ± 0.55% to 25.82% ± 1.04% gel contraction). Both pure ilomastat and ilomastat-CD at a concentration of 0.01 μM were less effective at inhibiting collagen gel contraction (51.79% ± 2.05% and 46.81% ± 2.12% gel contraction, respectively).

### In Vivo Distribution of Ilomastat

The ocular distribution of ilomastat in rabbit eyes was evaluated 4 hours after administration of ilomastat-CD solution (20% CD without HA) (100 μL, 1 mg/mL). The level of ilomastat in the aqueous humor was 37.15 ± 3 1.42 nM (*n* = 3). There was a negligible amount of ilomastat in the vitreous (0.07 ± 0.05 nM) (*n* = 4). The level of ilomastat in the sclera was 2-fold higher than in conjunctiva and cornea (Table). These levels are within the known therapeutic concentration range of ilomastat that would be expected by subconjunctival injection to treat fibrosis after glaucoma surgery.^6,7^ Both the conjunctiva and cornea displayed similar levels of ilomastat (Table). This result is promising as topical administration of ilomastat-CD could also be considered to treat corneal injury.

**Table:**
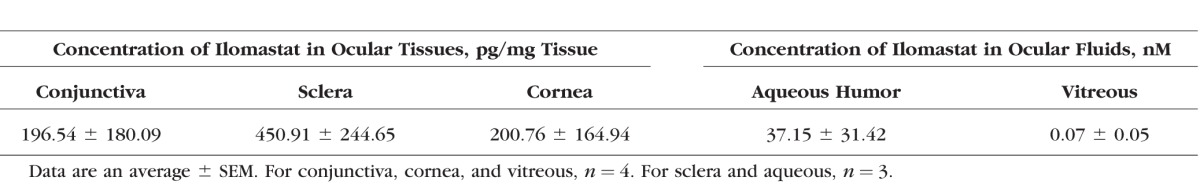
Level of Ilomastat in Ocular Tissues and Fluids

## Discussion

These results show that it is possible to formulate ilomastat by complexation with CD. Ilomastat has poor water solubility, and the solubility of ilomastat was improved by complexation with CD. Inflammation and fibrosis are the main cause of the failure of glaucoma filtration surgery (GFS).^43^ Ilomastat is a wide-spectrum MMPi that has shown improved GFS outcome in rabbits by repeated subconjunctival injection.^6,7^

CDs are widely used to improve the aqueous solubility of poorly soluble drugs.^16^ 2-Hydroxypropyl-β-cyclodextrin was selected to prepare a soluble form of ilomastat because this CD (1) is in eye drops for human use,^44,45^ (2) is nontoxic for topical ophthalmic use,^16,44,46^ and (3) has high water solubility (450 mg/mL).^47^ Several methods were investigated to prepare the ilomastat-CD complex, including (1) mixing ilomastat dissolved in aqueous tert-butanol (40% vol/vol) with an aqueous solution of CD, then freeze drying (the lyophilised powder was not soluble in water); (2) mixing a suspension of ilomastat with aqueous CD (the drug remained insoluble); and (3) using solid addition method (CD powder was added slowly to an aqueous suspension of ilomastat). This last method successfully solubilized ilomastat and maintained ilomastat solubility after freeze drying. Complexation of ilomastat with CD did not affect the in vitro activity of ilomastat. Several studies reported that complexation of drugs to CD did not impair their biological activities in the eye,^17,27,28^ probably due to a reversible equilibrium between free drug and drug-CD.^16^

The presence of CD might facilitate permeation of ilomastat through ocular tissues. CD binds to biological membranes and increases the permeability of the complexed drug.^48,49^ This was confirmed by (1) increased permeation of ilomastat through pig conjunctiva in vitro compared to pure ilomastat and (2) the presence of ilomastat in ocular tissues and fluids after topical administration of ilomastat-CD solutions. Cyclodextrin has been reported to increase permeability of drugs such as sirolimus,^50^ natamycin,^19^ tranilast,^51^ hesperidin,^52^ ketoconazole,^53^ pilocarpine,^25,54^ and thalidomide^26^ through ocular tissues after topical application. This is important, as much of the therapeutic effect for ocular drops depends on penetration into ocular tissues.

The biodistribution studies revealed that the concentration of ilomastat was within the therapeutic range (10–100 nM)^4,6,7^ in both ocular tissues (sclera and conjunctiva) and fluids (aqueous humor). These results suggest that the ilomastat-CD complex has the potential to be used as a topical modulator of ocular wound healing.

## Conclusions

Complexation of ilomastat with CD improved its solubility and permeability through ocular tissues with retention of its in vitro activity. In vivo biodistribution studies displayed the presence of ilomastat at a therapeutic concentration in sclera, conjunctiva, and aqueous humor. These promising results reveal that topical application of ilomastat might be successful to inhibit scarring following glaucoma surgery. More work needs to be conducted to establish the in vivo efficacy of the ilomastat-CD eyedrop.

## Supplementary Material

Supplement 1
