# Supplementary material for: An Ilomastat-CD Eye Drop Formulation to Treat Ocular Scarring
Source: Invest Ophthalmol Vis Sci. 2017 Dec;58(9):3425–31. doi: 10.1167/iovs.16-21377 (PMC5713897; doi:10.1167/iovs.16-21377)

Supplementary information

Table S1. Liquid chromatography gradient conditions for LC-MS analysis. FA is formic acid.

Time (min):	0.1% FA in Water (%)	0.1% FA in ACN (%)
0.0	95	5
0.5	95	5
3.0	5	95
4.0	5	95
4.2	95	5
7.0	95	5

Figure S1. Aqueous solubility of ilomastat in the presence of CD and PVP.

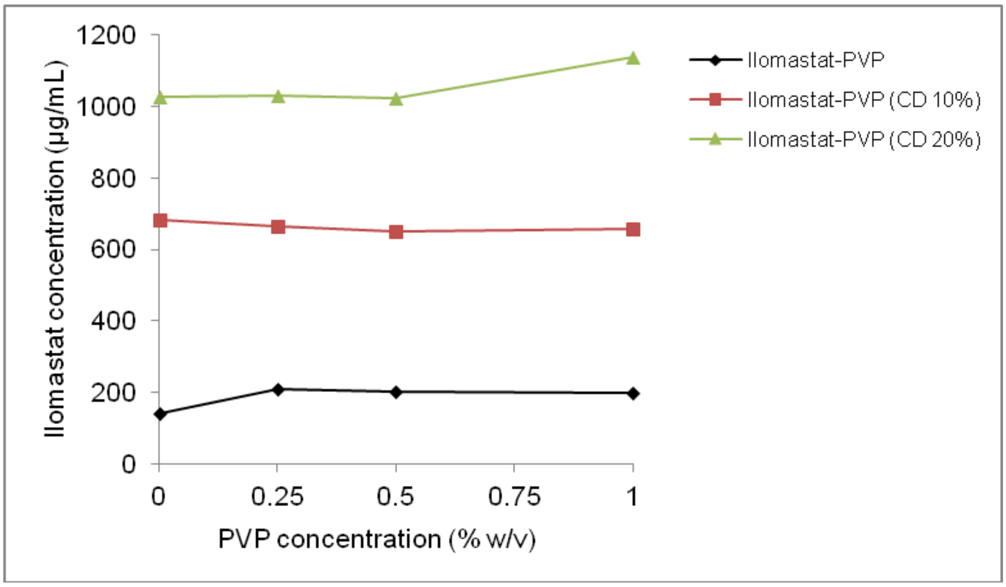

Supplement: Supplement 1 [file iovs-58-07-49_s01.pdf]
